# A Pregnancy and Postnatal RCT Among Women With Gestational Diabetes Mellitus and Overweight/Obesity: The PAIGE2 Study

**DOI:** 10.1210/jendso/bvae151

**Published:** 2024-09-11

**Authors:** Bridie J Kemp, Bronagh Kelly, Georgina Cupples, Olwen Fleck, Emma McAuley, Rachel M Creighton, Helen Wallace, Una Graham, Ciara Mulligan, Adele Kennedy, Chris C Patterson, David R McCance

**Affiliations:** Regional Centre for Endocrinology and Diabetes, Royal Jubilee Maternity Centre, Belfast Health and Social Care Trust, Belfast BT12 6BA, UK; Regional Centre for Endocrinology and Diabetes, Royal Jubilee Maternity Centre, Belfast Health and Social Care Trust, Belfast BT12 6BA, UK; Regional Centre for Endocrinology and Diabetes, Royal Jubilee Maternity Centre, Belfast Health and Social Care Trust, Belfast BT12 6BA, UK; Regional Centre for Endocrinology and Diabetes, Royal Jubilee Maternity Centre, Belfast Health and Social Care Trust, Belfast BT12 6BA, UK; Regional Centre for Endocrinology and Diabetes, Royal Jubilee Maternity Centre, Belfast Health and Social Care Trust, Belfast BT12 6BA, UK; Regional Centre for Endocrinology and Diabetes, Royal Jubilee Maternity Centre, Belfast Health and Social Care Trust, Belfast BT12 6BA, UK; Regional Centre for Endocrinology and Diabetes, Royal Jubilee Maternity Centre, Belfast Health and Social Care Trust, Belfast BT12 6BA, UK; Regional Centre for Endocrinology and Diabetes, Royal Jubilee Maternity Centre, Belfast Health and Social Care Trust, Belfast BT12 6BA, UK; Diabetes & Endocrinology, Ulster Hospital, South Eastern Health and Social Care Trust, Belfast BT16 1RH, UK; Endocrine and Diabetes, Antrim Area Hospital, Northern Health and Social Care Trust, Antrim BT41 2RL, UK; Centre for Public Health, School of Medicine, Dentistry and Biomedical Sciences, Queen's University Belfast, Belfast BT12 6BA, UK; Regional Centre for Endocrinology and Diabetes, Royal Jubilee Maternity Centre, Belfast Health and Social Care Trust, Belfast BT12 6BA, UK

**Keywords:** gestational diabetes mellitus, lifestyle, overweight, obesity, pregnancy, randomized controlled trial

## Abstract

**Objective:**

This study examined the influence of a pregnancy and postnatal multicomponent lifestyle intervention for women with gestational diabetes mellitus (GDM) and overweight/obesity from 6 weeks to 12 months postnatal. The primary outcome was weight at 12 months. Secondary outcomes included change in body mass index (BMI), waist circumference (WC) and fasting plasma glucose (FPG).

**Methods:**

The study involved 235 pregnant women with GDM and BMI ≥ 25 kg/m^2^ during pregnancy. Intervention components included an educational session, activity tracker (Fitbit), monthly phone calls, weekly motivational text messages, 12-week voucher for a commercial weight management organization and anthropometric, biochemical, and clinical measurements taken at 6 weeks, 6 months, and 12 months postnatal. The control group received routine local maternity care.

**Results:**

A mean weight change of −2.0 (SD 7.1) kg was observed in the intervention group compared with −0.6 (SD 8.0) kg in the control group, difference −1.4 (95% CI −4.4, 1.5) kg from 6 weeks to 12 months postnatal, but this was not statistically significant (*P* = .34). Neither were significant differences obtained for any secondary outcomes: BMI −0.6 (−1.6, 0.5) kg/m^2^, WC −1.0 (−5.1, 3.2) cm and FPG 0.07 (−0.15, 0.29) mmol/L.

**Conclusion:**

This lifestyle intervention among women with overweight/obesity and GDM resulted in a statistically nonsignificant 1.4 kg greater weight loss compared with routine care at 12 months postnatal. Further research is needed to understand how the different components of this lifestyle intervention might be better applied to elicit more successful results.

The obstetric and public health significance of gestational diabetes mellitus (GDM) (hyperglycemia with first onset or recognition during pregnancy) is now increasingly recognized [1]. The global pooled prevalence of GDM was estimated as 14% of pregnancies in 2021 [2], with some countries reporting GDM prevalence in up to 30% of pregnancies [3]. The association of GDM with an increased risk of adverse short-term and long-term maternal-fetal outcomes is now well established [4, 5]. Women with GDM have a ≥ 50% risk of developing GDM in a future pregnancy and a 10-fold increase in risk of developing type 2 diabetes, compared to women without GDM [6]. The risk is compounded by other risk factors, in particular maternal overweight/obesity, which together with GDM, increases the subsequent development of both GDM and type 2 diabetes [3].

A number of interventions seeking to prevent women from developing GDM have been reported [7, 8], together with interventions to treat women with GDM during pregnancy [9]. While a 2016 meta-analysis of 29 randomized controlled trials (RCTs) involving 11 487 pregnant women found that lifestyle modification before 15 weeks gestation could reduce the risk of developing GDM (relative risk [RR] = 0.80; 95% CI 0.66, 0.97) [10], individual studies have reported less successful results due to difficulty in recruiting high-risk women [11, 12].

Additionally, a systematic review of 15 studies (5502 women) and meta-analysis of 8 studies (1742 women) that explored the prevention of type 2 diabetes in women with previous GDM through different lifestyle interventions (mostly dietary and physical activity), found that only those trials intervening soon after delivery (< 1 year postnatal) significantly reduced the risk (RR = 0.61; 95% CI 0.40, 0.94; *P* = .02) [13]. Significant reductions with intervention relative to control were observed for weight: −1.07 (95% CI −1.43, −0.72) kg, body mass index (BMI): −0.94 (95% CI −1.79, −0.09) kg/m^2^, and waist circumference (WC): −0.98 (95% CI −1.75, −0.21) cm, with larger reductions in studies with the longest follow-up [13]. Of note, in some of the included studies, the review identified concerns regarding random sequence generation and allocation concealment [13]. The results, however, were congruent with the PAIGE pilot study published in 2018, a randomized lifestyle postnatal intervention to promote weight loss among 60 overweight women with previous GDM conducted between 6 weeks postnatal and 6 months postnatal [14]. The PAIGE study showed significantly greater weight loss in the intervention group at 6 months postnatal compared with usual care (mean [SD] 3.9 [7.0] kg vs 0.7 [3.8] kg, *P* = .02) [14]. Feedback from the women suggested they favored the multicomponent nature of the study; however, the sustainability of such a study over a longer period is unknown [14]. One recommendation from the PAIGE study feedback was that the intervention should begin before 6 weeks postnatal. This aligned with a recent systematic review that advocated the implementation of a lifestyle intervention both during and after pregnancy to produce successful results in reducing the health risks associated with GDM [15].

Informed by the PAIGE pilot study, the PAIGE2 study was a pragmatic lifestyle RCT intervention for women with overweight and obesity diagnosed with GDM during their most recent pregnancy to effect weight loss at 12 months postnatal compared with women receiving routine care.

## Methods

A protocol for the PAIGE2 study has been published elsewhere [16]. Briefly, the PAIGE2 intervention [14], was a pragmatic, cluster-randomized, controlled, lifestyle intervention trial consisting of 2 parallel arms based in the Royal Victoria Hospital in Belfast, Northern Ireland involving recruitment from 3 maternity centers in the Belfast, Northern and South Eastern Health and Social Care Trusts, involving 235 women with GDM (diagnosed using the IADPSG/2013 WHO criteria as in the PAIGE study [14]) and BMI ≥ 25 kg/m^2^ at their booking appointment (< 14 weeks gestation), recruited during pregnancy [16]. The primary outcome was maternal weight, and secondary outcomes were BMI, WC, and fasting plasma glucose (FPG), at 12 months postnatal.

Around 32 to 36 weeks gestation (time point 1; TP1), participants within each center were randomized in clusters according to the calendar week of their visit, using a randomization list stratified by center and restricted for balance. The list was generated in advance of the trial using the on-line tool Randomisation.com (https://randomisation.com/) and allocations were concealed from participants until they were entered in the trial.

The intervention group received a 1-hour educational session comprising information on the causes, consequences, difficulties, and implications of GDM and overweight/obesity, on a healthy nutritional approach to improving their lifestyle, and on the effectiveness of physical activity as an alternative treatment for the prevention of subsequent GDM and type 2 diabetes including recommendations, barriers, and strategies [16]. The control group received routine local maternity care and continued to receive routine follow-up care throughout the intervention.

From 6 weeks postnatal (time point 2: TP2) through 6 months postnatal (time point 3: TP3) until 12 months postnatal (time point 4: TP4), the intervention group also received monthly phone calls (including information of physical activity, barriers to achieving diet and physical activity goals, as well as suggested solutions), weekly motivational text messages, weekly step counts (using a complimentary activity device [Fitbit]), and were offered at least one complimentary voucher to attend the commercial weight management organization Slimming World (SW), for 3 months [16]. Three postnatal study visits at 6 weeks (TP2), 6 months (TP3), and 12 months (TP4) took place for all study participants to obtain anthropometric and clinical measurements, fasting blood samples, and to complete questionnaires regarding their health, wellbeing, and physical activity [16].

Women in the intervention group who completed TP4 (12 months postnatal) were invited to participate in focus groups to discuss their experience, as per protocol (described elsewhere) [17]. The CONSORT checklist for reporting RCTs was used to report the quantitative results (ie, the primary, secondary, and other outcomes) from the PAIGE2 study [16, 18].

Primary and secondary outcomes at 12 months postnatal, relative to those at baseline (6 weeks postnatal), were compared between the intervention and control groups using analysis of covariance to produce an estimate of the intervention effect, with 95% CI and *P* value, adjusting for any differences in the outcome at baseline [16]. Analyses were additionally adjusted for center and corrected for the clustered nature of the randomization. For convenient interpretation, findings were also summarized as changes from baseline. These analyses were repeated with multiple imputation of missing values to assess any possible role that nonresponse might have on the findings. Other comparisons between groups at the 6-week, 6-month, and 12-month time points were made using independent sample *t* tests (quantitative variables) and chi-squared tests (categorical variables). For International Physical Activity Questionnaire results (IPAQ), median (interquartile range [IQR]) rather than mean (SD) were used because of the heavy skew in their distribution. Differences were tested by Mann-Whitney U tests. In accordance with IPAQ recommendations, outliers with implausibly large exercise durations were excluded. Using the variability of weight changes observed in the PAIGE pilot trial, it was estimated that a trial of 340 women should yield 200 evaluable subjects (100 in each of the intervention and control groups) which would be sufficient to detect a 3-kg difference in mean maternal weight loss at 12 months after delivery with > 90% power using a 2-tailed test at the 5% significance level [16].

The study conformed with the principles outlined in the Declaration of Helsinki [19]. Research governance approval was gained from the Belfast Health and Social Care Trust (18/NI/0228), and ethical approval granted from the Office for Research Ethics Committees Northern Ireland. Participants provided written consent prior to participating in the PAIGE2 study.

## Results

**Figure 1.**
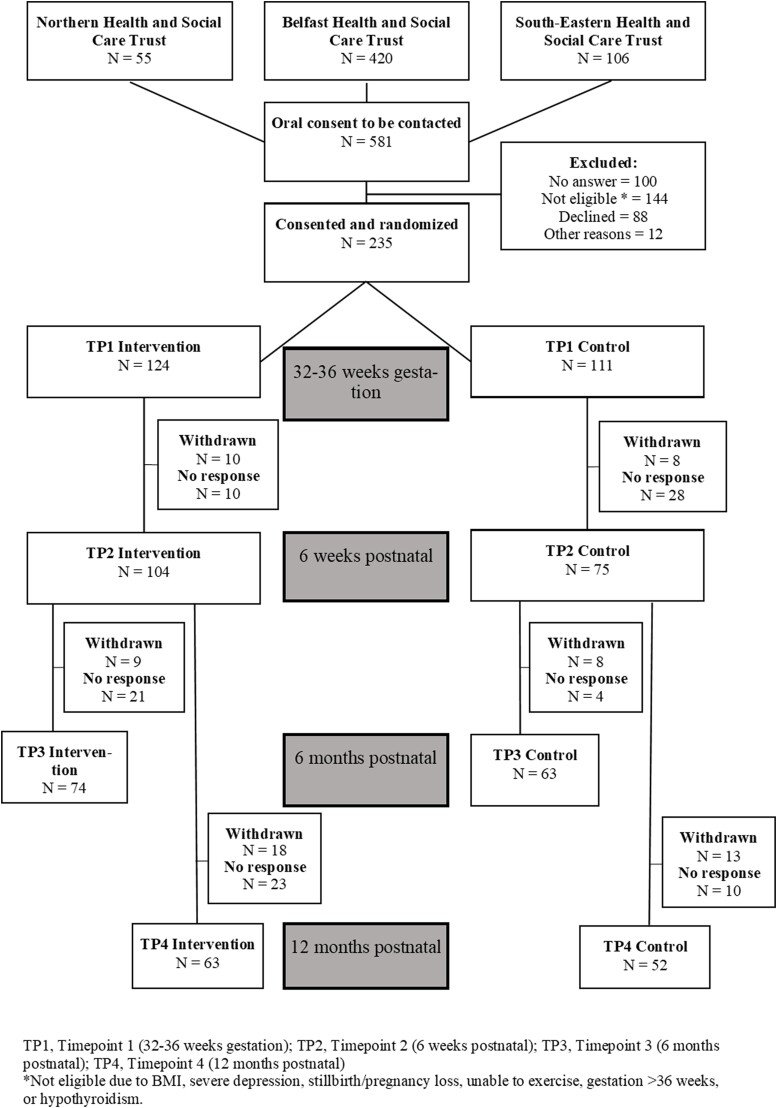
Depicts the PAIGE2 second study participant flowchart.

Of the 581 women screened for eligibility across the 3 centers during recruitment, 235 women were randomly assigned either to the intervention (n = 124) or the control group (n = 111) at TP1. In total, 104 women in the intervention group and 75 women in the control group continued in the study at TP2 (6 weeks postnatal), 74 and 53 women respectively at TP3 (6 months postnatal), and finally 63 and 52 women respectively at TP4 (12 months postnatal), giving retention rates at TP4 of 50.8% and 46.8% for the intervention and control groups (Fig. 1).

Demographic data at time of recruitment during pregnancy (TP1) and clinical/biochemistry data at 6 weeks postnatal (TP2) are detailed in Table 1 and compared with those women who dropped out during follow-up in Table 2. Other than differences in marital status and FPG at recruitment, there were no other differences between the completers and dropouts. At the time of recruitment, the mean (SD) age was 30.6 (4.9) years in the intervention group and 31.0 (5.5) years in the control group. Most participants in both groups were of White ethnic background (97.4%), employed (75.7%), and were either married or cohabiting (70.6%). For 37.9% of all the women, this was their first child. Rates of breastfeeding at hospital discharge were 30.8% for exclusive breastfeeding and 19.2% for mixed breast and bottle feeding, indicating that half the women (50.0%) were not breastfeeding at discharge.

**Table 1.** Demographic data at time of recruitment during pregnancy (TP1) and clinical/biochemistry data at 6 weeks postnatal (TP2)

	Intevention		Control		
	n	Mean ± SD or (%)	n	Mean ± SD or (%)	*P*
Demographic data at time of recruitment (TP1)					
Age (y)	124	30.6 ± 4.9	111	31.0 ± 5.5	.48
White ethnicity	120/123	(98%)	109/111	(98%)	1.00
Employed, full or part-time	97/113	(86%)	81/100	(81%)	.44
Married or cohabiting	93/114	(82%)	73/99	(74%)	.23
Nulliparous	45/124	(36%)	44/111	(40%)	.69
Worst fifth of deprivation	45/124	(36%)	38/111	(34%)	.85
Clinical/biochemistry data at 6 weeks postnatal (TP2)					
Breast feeding, fully or partially***^a^***	62/123	(50%)	55/111	(50%)	1.00
Weight (kg)	104	92.3 ± 15.5	75	93.3 ± 13.5	.68
BMI (kg/m^2^)	104	34.6 ± 5.1	75	34.5 ± 4.2	.89
Waist circumference (cm)	86	108.5 ± 12.0	61	108.2 ± 12.2	.87
Systolic BP (mmHg)	62	122.9 ± 14.2	42	125.3 ± 10.8	.37
Diastolic BP (mmHg)	62	79.1 ± 8.9	42	79.1 ± 8.6	1.00
Fasting plasma glucose (mmol/L)	66	4.99 ± 0.45	44	5.04 ± 0.36	.50
Cholesterol (mmol/L)	65	5.05 ± 0.94	44	5.22 ± 0.93	.36
Triglycerides (mmol/L)	65	1.30 ± 0.68	44	1.49 ± 0.71	.17
HDL cholesterol (mmol/L)	65	1.39 ± 0.34	44	1.40 ± 0.32	.88
LDL cholesterol (mmol/L)	65	3.07 ± 0.77	44	3.14 ± 0.83	.65
Non-HDL cholesterol (mmol/L)	65	3.66 ± 0.92	44	3.82 ± 0.94	.38
Cholesterol/HDL cholesterol ratio	65	3.81 ± 1.03	44	3.90 ± 1.01	.66

Abbreviations: BP, blood pressure; HDL, high density lipoprotein; LDL, low density lipoprotein.

^*a*^At time of discharge.

**Table 2.** Demographic data at time of recruitment during pregnancy (TP1) and clinical/biochemistry data at 6 weeks postnatal (TP2) compared in completers and dropouts

	Completers		Dropouts		
	n	Mean ± SD or (%)	n	Mean ± SD or (%)	*P*
Demographic data at time of recruitment (TP1)					
Age (y)	115	31.4 ± 5.1	120	30.2 ± 5.2	.08
White ethnicity	114/115	(99%)	115/119	(97%)	1.00
Employed, full or part-time	99/113	(88%)	79/100	(79%)	.13
Married or cohabiting	96/112	(86%)	70/98	(71%)	.02*
Nulliparous	41/115	(36%)	48/120	(40%)	.58
Worst fifth of deprivation	38/115	(33%)	45/120	(38%)	.56
Clinical/biochemistry data at 6 weeks postnatal (TP2)					
Breast feeding, fully or partially***^b^***	62/115	(54%)	55/119	(46%)	.30
Weight (kg)	115	93.1 ± 14.4	64	91.9 ± 15.3	.60
BMI (kg/m^2^)	115	34.5 ± 4.4	64	34.4 ± 5.0	.91
Waist circumference (cm)	100	109.2 ± 12.1	47	106.6 ± 11.7	.22
Systolic BP (mmHg)	66	123.7 ± 9.9	38	124.3 ± 17.2	.82
Diastolic BP (mmHg)	66	79.6 ± 7.9	38	78.2 ± 10.1	.44
Fasting plasma glucose (mmol/L)	72	4.94 ± 0.38	38	5.16 ± 0.45	.***01***^*a*^
Cholesterol (mmol/L)	71	5.10 ± 0.97	38	5.15 ± 0.87	.78
Triglycerides (mmol/L)	71	1.38 ± 0.71	38	1.37 ± 0.69	.94
HDL cholesterol (mmol/L)	71	1.37 ± 0.33	38	1.43 ± 0.32	.34
LDL cholesterol (mmol/L)	71	3.10 ± 0.83	38	3.09 ± 0.72	.94
Non-HDL cholesterol (mmol/L)	71	3.73 ± 0.97	38	3.72 ± 0.85	.95
Cholesterol/HDL cholesterol ratio	71	3.91 ± 1.06	38	3.73 ± 0.93	.40

Abbreviations: BP, blood pressure; HDL, high density lipoprotein; LDL, low density lipoprotein.

^*a*^ *P* < .05.

^*b*^At time of discharge.

At 12 months postnatal, the mean weight change of the women in the intervention and control groups was −2.0 (7.1) kg and −0.6 (8.0) kg, respectively (Table 3). Analysis of covariance provided an estimated intervention effect of −1.4 (95% CI −4.2, 1.3) kg; *P* = .34. There was also no significant difference in the secondary outcomes of BMI, WC, and FPG in the intervention vs control group at 12 months postnatal (Table 3). Comparison of weight change between TP2 and TP3 also showed no significance between the intervention and control groups (−1.4 [5.3] kg vs 0.0 [5.6] kg, respectively), with an estimated intervention effect of −1.4 (95% CI −3.5, 0.7) kg; *P* = .18. There was also no significant difference in the secondary outcomes between these time points. Imputation of missing data did not materially alter any of these results.

**Table 3.** Primary and secondary outcomes at 12 months (TP4) compared in the intervention and control groups with adjustment for results at 6 weeks postnatally (TP2)

	Treatment								Difference (95% CI) P*^a^*
	Intervention				Control				
	n	Initial	Final	Change	n	Initial	Final	Change	
Weight (kg)	63	93.6 (14.5)	91.6 (16.0)	−2.0 (7.1)	52	92.5 (14.5)	91.9 (15.9)	−0.6 (8.0)	−1.4 (−4.4,1.5) .34
BMI (kg/m^2^)	63	34.6 (4.6)	33.9 (5.3)	−0.7 (2.6)	52	34.4 (4.4)	34.2 (5.1)	−0.2 (2.9)	−0.6 (−1.6, 0.5) .31
Waist circumference (cm)	47	108.5 (10.1)	105.9 (11.8)	−2.6 (10.5)	36	108.1 (12.9)	106.5 (13.6)	−1.5 (9.4)	−1.0 (−5.1, 3.2) .65
Fasting plasma glucose (mmol/L)	33	4.92 (0.40)	5.10 (0.54)	0.18 (0.60)	24	5.00 (0.37)	5.05 (0.34)	0.06 (0.38)	0.07 (−0.15, 0.29) .52

Values are mean (SD).

Abbreviation: BMI, body mass index.

^*a*^ANCOVA adjusting for outcome value at 6 weeks postnatally and center and correcting for cluster randomization.

There were no statistically significant differences between the groups in the information gleaned from the several maternal questionnaires at TP2 and TP4, including IPAQ, Risk Perception Survey for Developing Diabetes (RPSDD), 12-Item Short Form Health Survey (SF-12v2), and motivation for change (Table 4). The only statistically significant result was for one question relating to the difference between mother's risk perception for developing diabetes as being within her personal control between TP2 and TP4, but this finding is difficult to interpret in the light of the multiple tests performed.

**Table 4.** Questionnaire result changes from 6 weeks to 12 months postnatal (TP2 to TP4)

	Treatment						*P*
	Intervention			Control			
	n	Mean	SD	n	Mean	SD	
Risk Perception for Developing Diabetes—Personal control^*a*^	22	0.17	0.35	28	−0.09	0.50	.04
Risk Perception for Developing Diabetes—Optimistic bias	22	0.23	0.78	28	−0.09	0.51	.09
Risk Perception for Developing Diabetes—Knowledge score	22	0.68	1.62	28	0.18	1.52	.26
Risk Perception for Developing Diabetes—Benefits vs barriers	17	−0.12	0.29	22	−0.23	0.70	.55
Motivation to Change—How important are lifestyle changes …*^b^*	24	−0.37	1.31	25	−0.60	1.44	.57
Motivation to Change—How confident you can make changes … *^c^*	24	−0.29	2.40	25	−0.60	2.29	.65
Exercise Self Efficacy—Making time	23	−0.32	0.85	28	−0.25	0.79	.76
Exercise Self Efficacy—Resisting relapse	23	−0.66	1.12	28	−0.40	0.76	.32
Short Form 12—Physical component summary	20	5.6	8.2	21	2.2	7.9	.18
Short Form 12—Mental component summary	20	−3.2	13.9	21	−4.3	13.0	.79

	Treatment				*P*
	Intervention		Control		
	n	Median (IQR)	n	Median (IQR)	
IPAQ—Total MET minutes (per week)	21	199 (−1722, 2670)	23	1131 (−576, 3856)	.31
IPAQ—Total Sitting Time (minutes per week)	19	0 (−840, 615)	20	−270 (−695, 488)	.86

Abbreviations: IPAQ, International Physical Activity Questionnaire; IQR, interquartile range.

^*a*^ *P* < .05.

^*b*^How important is it to you that you make changes to your lifestyle to help you lose weight and reduce your risk of type 2 diabetes in the future?

^*c*^How confident are you that you can make the necessary changes to your lifestyle to help you to lose weight and reduce your risk of type 2 diabetes in the future?

Only 41 (33%) of the women in the intervention group availed of the free 12-week complimentary SW voucher, and, of these women, 31 completed the trial. A comparison of these 31 who attended (and with 12-month endpoint data) compared with the 32 who did not, showed a mean weight change between TP2 and TP4 of −3.1 kg vs −1.0 kg (*P* = .24). In addition, no statistically significant differences in BMI or WC changes were observed from TP2 to TP4, or in weight, BMI, and WC changes from TP2 to TP3.

There were no statistically significant differences between mothers’ mean daily step count recorded by the activity tracking device (Fitbit) in the intervention group either between TP2 and TP3 (n = 35; 270 steps per day increase; 95% CI −43, 971; *P* = 0.44), or between TP2 and TP4 (n = 28; 2 steps per day increase; 95% CI −1093, 1096; *P* = 1.00).

All women in the intervention group were asked to complete an end of study feedback questionnaire. In total, 53 (42.7%) of the 124 responded. Most women (58.5%) preferred to interact with the study by a mixture of face-to-face and virtual communication. When questioned regarding the most motivational aspects of the intervention, 92.5% considered the 1-hour education session at TP1 useful, 71.7% were satisfied with the frequency of motivational text messages they received throughout the intervention, 83.0% were satisfied with the number of phone calls they received throughout the intervention, 73.6% were satisfied with the timing of the study visits throughout the intervention. In response to questions about different aspects of the intervention, 92.5% strongly agreed or agreed that the Fitbit they received as part of the intervention helped to motivate them. Finally, 84.9% considered that, as a result of taking part in the study, they had made lifestyle changes.

## Discussion

This report describes the results of the PAIGE2 study, an RCT lifestyle intervention trial for women with overweight and obesity diagnosed with GDM during their most recent pregnancy to facilitate weight loss between 6 weeks and 12 months postnatal. The results showed a 1.4-kg difference in weight between the intervention and control group at 12 months postnatal, but this was not statistically significant. In addition, there were no significant differences in BMI, WC, or FPG between the groups.

Our results differ from those of the PAIGE pilot study, which followed women for 6 months [14], and also a meta-analysis of 1742 women, which reported that trials intervening less than 1 year after delivery [13] were effective in achieving a reduction in weight and BMI. Informed by the PAIGE pilot study and concordant with recommendations from systematic reviews [9, 13, 14], the PAIGE2 intervention commenced during pregnancy with a 1-hour education session, but this did not appear to influence the results, albeit PAIGE2 involved a longer follow-up than the PAIGE pilot. On inquiry, mothers in the intervention group expressed satisfaction with several specific intervention components, such as the motivational texts and the phone calls and were most motivated by the Fitbit used throughout the intervention.

Of the 41 women in the intervention group who availed of the complimentary 12-week SW voucher, 31 completed the intervention and a mean weight loss of −3.1 kg was observed. This compares with a 10.26 kg weight loss for 98 intervention women at 12 months postnatal reported in an RCT that used lifestyle information and SW to support postnatal weight loss in women who were either overweight at their booking appointment or had experienced excessive gestational weight gain at 36 weeks gestation [20]. However, unlike PAIGE2, these latter women were not diagnosed with GDM during pregnancy, and their weight at 12 months was compared to their weight at the end of their pregnancy, not at 6 weeks postnatal. In both studies, participation in SW appeared to result in weight loss, although not all women availed of the opportunity afforded to them. Future studies should consider the structure and information provided by commercial weight management organizations such as SW to facilitate postnatal weight loss and whether this needs to be modified in subjects with previous GDM as distinct from obesity per se. A solution may be a more collaborative approach between researchers and the commercial provider to ensure a consistent approach/advice is provided.

The most positive feedback from the mothers related to the activity tracker (Fitbit) which the women received during pregnancy and continued to use until 12 months postnatal, accompanied by motivational text messages from the research team regarding their weekly step counts. A recent systematic review and meta-analysis found that Fitbits were helpful in promoting healthier lifestyle behaviors in terms of both increasing physical activity levels and weight loss [21]. In PAIGE2, however, we did not observe any statistically significant changes in weight between the groups nor was there an increase in daily steps between TP2 and TP4 with Fitbit use, despite weekly monitoring via messages. In a recent systematic review and meta-analysis, text message interventions were found to lead to greater physical activity [22], and in an RCT involving text messages to help women with overweight and obesity lose weight after childbirth [23]. More research is needed to understand if the weekly monitoring of lifestyle behaviors and regular surveillance of weight, step counts, and nutritional intakes could help future studies elicit more positive results regarding weight loss for mothers with overweight or obesity and GDM to help reduce their future risk of GDM, obesity and type 2 diabetes.

## Strengths and Limitations

There were several strengths to this study. First, this study was informed by the PAIGE pilot study that showed significant weight change albeit at a shorter period of follow-up (6 months). Secondly, we believe the structure of the PAIGE2 lifestyle intervention, which commenced during and continued after pregnancy in women with a recent diagnosis of GDM and overweight/obesity, has not been reported previously, where interventions either during or after pregnancy have been reported. Thirdly, PAIGE2 was a multicenter RCT, covering 3 of 6 Health and Social Care Trusts in Northern Ireland, thus widening the scope of women who could participate across the country and increasing the generalizability of the trial.

However, PAIGE2 had also several limitations. First, the COVID-19 pandemic began at the start of the PAIGE2 study. This necessitated pragmatic changes to the running of the study (eg, increasing use of virtual methods of communication including SW, and reliance on self-reported weight and WC assessments) which required ethical amendments and in general, delayed recruitment of participants, and may also have indirectly affected the study due to changes in lifestyle habits globally [24]. Second, during the pandemic, staff originally recruited to work on the PAIGE2 study were redeployed, which impacted on the study for a time. Third, the study was designed to recruit subjects from cross-border regions in Northern Ireland and the Republic of Ireland, but unfortunately, due to differences in Research and Development structures across the 2 regions, together with pragmatic changes to the criteria for diagnosing GDM due to the COVID-19 pandemic, the 3 Republic of Ireland centers had to withdraw from the study. Fourth, due to 2 separate cyber-attacks made against the Belfast Health and Social Care Trust, some communications were delayed while others ceased completely resulting in a significant delay in starting the study. Finally, related to the external pressures detailed above, recruitment to PAIGE2 was lower than planned, and greater variability in the control group weight changes and higher attrition (compared with the PAIGE pilot study), resulted in loss of statistical power. However, a retrospective power calculation showed that, although our study had less than 50% power to detect the originally proposed 3 kg differential weight loss between intervention and control groups, it had almost 80% power to detect a 4-kg differential weight loss and over 90% power to detect a 5-kg differential weight loss.

Overall, it is difficult to quantify to what extent the negative findings are due to the effects of the COVID-19 pandemic verses a true negative intervention. Undoubtedly COVID-19 had a major impact both on subjects’ willingness to take part and to attend for review which persisted for many months following the cessation of lockdown and resulted in us exploring all other “virtual” means of contact/education with considerable success, but face-to-face visits (and statistical power) were unquestionably compromised. Equally, our endeavor to facilitate the women by phone and text messages did not necessarily result in participant retention in the study and the provision of complimentary complementary passes to SW and activity devices were only utilized by a minority of participants. Our feedback questionnaires indicated that women approved of the contents of the PAIGE2 multicomponent intervention, but this feedback was obtained from those who participated. In our pilot study, women were recruited postnatally and studied at the time of attendance for their routine care 6 weeks postnatal oral glucose tolerance test, which undoubtedly improved the collection baseline metrics. The longer duration of PAIGE2 (12 months vs our 6 months PAIGE pilot) may also have been relevant and echoes the problem of attrition with longer studies described in the literature. The challenges and competing demands on these women in the postnatal period remain a perennial challenge to lifestyle intervention.

Despite these various challenges, the study was carried out to the highest possible standards, was granted a year's extension to complete by the funders/sponsor, and resulted in considerable innovation to maintain contact with the women and to support them in their postnatal health and lifestyle behaviors.

The PAIGE2 study did not show any statistically significant weight changes between the intervention and control group at 12 months postnatal following a lifestyle intervention that occurred both during and after pregnancy among women with GDM and overweight/obesity. More research is required to understand how the various intervention components might be better applied within the study to elicit more successful results.

## Data Availability

Some or all datasets generated during and/or analyzed during the current study are not publicly available but are available from the corresponding author on reasonable request.

## Prior Presentation

An abstract of this article was presented at the annual meeting of the European Diabetes in Pregnancy study Group in Poznan, Poland in September 2023.

## Clinical Trial Information

ClinicalTrials.gov registration no. NCT04579016.

## Abbreviations

BMI: body mass index
FPG: fasting plasma glucose
GDM: gestational diabetes mellitus
IPAQ: International Physical Activity Questionnaire
RCT: randomized controlled trial
RR: relative risk
SW: Slimming World
TP1: time point 1 (32-36 weeks gestation)
TP2: time point 2 (6 weeks postnatal)
TP3: time point 3 (6 months postnatal)
TP4: time point 4 (12 months postnatal)
WC: waist circumference
